# The Role of PTPN22 C1858T Gene Polymorphism in Diabetes Mellitus Type 1: First Evaluation in Greek Children and Adolescents

**DOI:** 10.1155/2013/721604

**Published:** 2013-07-15

**Authors:** Styliani Giza, Antonios Goulas, Emmanouela Gbandi, Smaragda Effraimidou, Efimia Papadopoulou-Alataki, Maria Eboriadou, Assimina Galli-Tsinopoulou

**Affiliations:** ^1^4th Department of Pediatrics, Faculty of Medicine, Aristotle University of Thessaloniki, Papageorgiou General Hospital, Ring Road Nea Efkarpia, 56403 Thessaloniki, Greece; ^2^1st Department of Pharmacology, Faculty of Medicine, Aristotle University of Thessaloniki, 54124 Thessaloniki, Greece; ^3^Department of Hematology, Papageorgiou General Hospital, Ring Road Nea Efkarpia, 56403 Thessaloniki, Greece

## Abstract

Type 1 diabetes mellitus (T1DM) is an autoimmune multifactorial disease. Protein tyrosine phosphatase nonreceptor type 22 (PTPN22) gene encodes lymphoid-specific tyrosine phosphatase (Lyp), an inhibitor of T cell activation. PTPN22 C1858T polymorphism was associated with T1DM in populations of Caucasian origin. The aim of this study was the investigation for the first time of the association of PTPN22 C1858T polymorphism with T1DM in Greek population. We studied 130 children and adolescents with T1DM and 135 healthy individuals of Greek origin. The polymorphism was genotyped using polymerase chain reaction with restriction fragment length polymorphism. C1858T and T1858T genotypes as well as 1858T allele were found more frequently in patients (10.8% and 5.8%, resp.) than in healthy individuals (5.9% and 3.0%, resp.) but at non statistically significant level. There was no statistically significant association found with gender, age at diagnosis, severity of onset, history of Hashimoto thyroiditis or family history of T1DM. Increased frequency of 1858T allele in patients than in controls, implying a probable association, agrees with results of similar studies on other populations. The inability to find a statistically significant difference is probably due to the decreased frequency of minor allele in Greek population, indicating the need for a larger sample.

## 1. Introduction

Type 1 diabetes mellitus (T1DM) is characterized by autoimmune destruction of insulin-producing beta cells of pancreatic islets by CD4^+^ cytotoxic T lymphocytes with the contribution of CD8^+^ helper T lymphocytes, which leads to permanent impairment of glucose metabolism. It is considered a multifactorial disease, requiring the interaction of genetic and environmental factors in triggering autoimmunity [1]. Although the exact pathogenesis of T1DM is unknown, several gene loci involved in disease outbreak have been identified. Among these, genes of human leukocyte antigen (HLA) class II, insulin (INS), cytotoxic T lymphocyte antigen-4 (CTLA-4), and protein tyrosine phosphatase non-receptor type 22 (PTPN22) play a key role [2].

PTPN22 gene is located on chromosome 1p13.3-p13.1 and encodes the lymphoid-specific tyrosine phosphatase (Lyp), an important inhibitor of the activation and proliferation of T lymphocytes. Downregulation is achieved through interaction of Lyp with the C-terminal Src tyrosine kinase (Csk), which suppresses T cell receptor (TCR) signaling [3]. A single nucleotide polymorphism (SNP) at position 1858 (rs 2476601) of the encoding sequence of PTPN22 gene, consisting of the substitution of cytosine by thymine, results in mutation of arginine to tryptophan at codon 620 of Lyp. The mutation disturbs the Lyp-Csk interaction, leading to uncontrolled TCR signaling and inappropriate prolonged activation of T lymphocytes, which induces and maintains autoimmunity. Although Lyp is a known inhibitor of TCR signaling, the regulatory mechanism still remains under investigation. Therefore, the study of PTPN22 C1858T polymorphism is of enormous clinical and therapeutic interest, as Lyp could be the target of intervention against triggered autoimmunity. Nowadays, research focuses on factors which inhibit cellular function and more specifically dephosphorylation of TCR activation-dependent kinases induced by Lyp [4].

For the first time, Bottini et al. [5] reported that 1858T allele was observed more frequently in patients with T1DM compared to healthy individuals from North America and Sardinia. Since then, studies have investigated the association of PTPN22 C1858T polymorphism with T1DM in various populations. The 1858T allele has been reported to be associated with T1DM in populations from Italy [6, 7], Germany [8, 9], Denmark [10], North America [11, 12], United Kingdom [13, 14], Spain [15] Ukraine [16], Estonia [17], Netherlands [18], Finland [19], France [20], Croatia [21], Norway [22], Poland [23], Russia [24], Colombia [25], Czech Republic and Azerbaijan [26], and Brazil [27] but not from India [28]. This allele has been associated with other autoimmune diseases such as systemic lupus erythematosus (SLE) [29], rheumatoid arthritis (RA) [30], juvenile idiopathic arthritis [31], multiple sclerosis [32], autoimmune thyroid diseases [33], psoriasis [34], and Addison disease [35] but not with inflammatory bowel disease [36].

In Greece, two studies have been conducted in populations from Crete. The minor allele was not associated with psoriasis in the first study [37], while it was associated with SLE but not with RA in the second one [38]. Given the fact that the frequency of minor allele varies between populations and that ongoing studies are important to confirm previous associations, the aim of this study was to assess the role of PTPN22 C1858T polymorphism in children and adolescents of Greek origin with T1DM.

## 2. Materials and Methods

This is a case-control study in a population of Greek origin. We studied 130 children and adolescents (73 males, 57 females) with T1DM (average age at entry to the study 11.47 ± 3.72 years, range 0.83–19.21) and 135 healthy subjects (72 male, 63 females) without a family history of T1DM. In order to minimize genetic heterogeneity, we included unrelated individuals with a history of at least three generations of Greek origin. Patients are followed up at the Pediatric Diabetes Outpatient Unit, 4th Department of Pediatrics, Faculty of Medicine, Aristotle University of Thessaloniki, Papageorgiou General Hospital. All patients were diagnosed with T1DM before the age of 15 years and were insulin dependent. Parameters included in the statistical analysis were gender, age at the entry to the study, age at diagnosis, severity of onset (presence or absence of ketoacidosis), personal history of Hashimoto thyroiditis (HT), and family history of T1DM of at least one first-degree relative. Control group consisted of children and adolescents older than 10 years, staff of Papageorgiou General Hospital, and students of the Medical Faculty of Aristotle University of Thessaloniki. Before entering the study, an informed consent was obtained by adult participants and parents or guardians of individuals younger than 18 years. The research protocol was declared at the service of ClinicalTrials.gov and approved by the Ethics Committee of Faculty of Medicine of Aristotle University of Thessaloniki. The study was conducted according to the criteria of the Declaration of Helsinki.

The method includes four stages: (a) genomic isolation of deoxyribonucleic acid (DNA), (b) polymerase chain reaction (PCR), (c) incubation of PCR products with restriction enzymes, and (d) electrophoresis. Whole blood was collected in a sterile vial containing anticoagulant ethylenediaminetetraacetic acid (EDTA). DNA was isolated using the QIAamp DNA Blood Mini Kit (QIAGEN Inc., CA, USA) according to the instructions of the manufacturer and was stored at −20°C. The piece of PTPN22 gene including polymorphism at position 1858 was amplified with specifically designed primers, PTPN22 forward 5-ACTGATAATGTTGCTTCAACGG-3 and PTPN22 reverse 5-TCACCAGCTTCCTCAACCAC-3. PCR products were incubated with the restriction enzyme RsaI (Fermentas, Germany) at 37°C overnight. The enzyme cuts C allele into two fragments of 172 base pairs (bp) and 46 bp, while T allele remains intact (218 bp). Electrophoresis of the products of digestion in 2.3% agarose gel followed.

Data were analyzed using the IBM program SPSS Statistics 19. Expected and observed frequencies of genotypes and alleles in patients and controls were compared in 2 × 3 and 2 × 2 tables, respectively, according to the Hardy-Weinberg equilibrium. The differences in frequencies of genotypes and alleles between patients and controls and between patient subgroups based on qualitative variables were analyzed by ×^2^ or Fisher exact test. Normal distribution of quantitative variables was examined using the Kolmogorov-Smirnov test. Normally distributed mean age at diagnosis was analyzed according to genotype analysis using analysis of variance (ANOVA). The level of statistical significance was defined at *P* < 0.05.

## 3. Results

In this study the distribution of genotypes in patients and controls did not differ significantly from the expected Hardy-Weinberg equilibrium (*P* = 0.605 and *P* = 0.930, resp.). The 1858T allele was detected more frequently in patients (5.8%) compared to controls (3.0%) but at nonstatistically significant level (*P* = 0.137, odds ratio, (OR)  =  2.00, and confidence interval (CI)  =  0.83–4.81). The T1858T genotype showed a reduced incidence in patients (0.8%) and was not detected in controls. The C1858T genotype was significantly more frequent in patients (10.0%) compared to controls (5.9%), but a statistically significant difference was not found. Because of the low frequency of 1858T allele, the C1858T and T1858T genotypes were grouped for statistical analysis. The C1858T and T1858T genotypes were detected more frequently in patients (10.9%) than in healthy subjects (5.9%) but not in a statistically significant level (Table 1).

There was a trend towards higher frequency of C1858T and T1858T genotypes in males. The frequency of C1858T and T1858T genotypes was 12.3% in males versus 6.9% in females in the patient group, while 8.8% versus 4.8%, respectively, in the control group (Table 2). However, the distribution of C1858T and T1858T genotypes carrying the predisposing allele did not differ significantly between males and females in the patient group as well as compared with the control group (Table 3).

The mean age at diagnosis was 7.35 ± 3.73 years (range 0.57–14.59). Statistical analysis after grouping patients according to age at diagnosis found that the PTPN22 C1858T polymorphism had the same effect in patients with early onset (age at diagnosis ≤10 years) as well as to those with late onset (age at diagnosis >10 years) of T1DM. The mean age at diagnosis was similar in patients with C1858T and T1858T genotypes as well as in those with C1858C genotype (7.68 and 7.31, years resp., *P* = 0.723). Furthermore, there was a tendency for the 1858T pubertal carriers to be more susceptible to T1DM than prepubertal (*P* = 0.361).

Investigating the possible association of the PTPN22 C1858T polymorphism with more severe clinical manifestation at diagnosis, assessed by the presence or absence of ketoacidosis, patients with C1858T and T1858T genotypes were diagnosed with ketoacidosis more often (11.5% versus 7.7%) but not in a statistically significant higher rate.

Moreover, we investigated the role of PTPN22 C1858T polymorphism in autoimmunity. Among C1858T and T1858T genotype carriers, HT presented with marginally increased incidence (14.8% T1DM + HT versus 9.7% T1DM only). We did not find statistically significant association of PTPN22 C1858T polymorphism with a positive family history of T1DM. The distribution of PTPN22 C1858T polymorphism in patients according to the analyzed parameters is shown in Table 3.

## 4. Discussion

In this case-control study, the role of PTPN22 C1858T polymorphism was investigated for the first time in children and adolescents of Greek origin with T1DM. The first report of the association of PTPN22 C1858T polymorphism with T1DM by Bottini et al. [5] was confirmed by several association studies [5–26]. This finding was reinforced by recently published meta-analyses which suggest that PTPN22 C1858T polymorphism may contribute to the predisposition of the T1DM especially in populations of Europe and America. However, the investigators conclude that there is still need for more studies to support these findings [39–44]. In the present study population, although increased 1858T allele frequency was confirmed in patients with T1DM, the result was not statistically significant. However, OR of 1858T versus 1858C allele in patients compared to controls was similar to that of other studies [39]. 

The studies conducted in different populations can indirectly reveal an interesting feature of PTPN22 C1858T polymorphism, the large geographical variation in the frequency of the 1858T allele. There is a tendency for increased frequency of the 1858T allele in healthy Caucasian populations in Europe (from southern to northern). More specifically, its frequency was found to be 2% in Italy, 6% in Spain, 12% in Sweden, and 15.5% in Finland. However, it seems to be almost absent in populations from Asia and Africa [40]. In Greece, the 1858T allele frequency was found to be low (2.69–2.91%) in 3 independent groups of healthy individuals from Crete [37, 38]. A similar incidence (3.0%) was found in the control group of the study population. Therefore, the main comparison standard for the outcome of this study is more appropriate to be published studies involving populations of Southern Europe where the frequency of the 1858T allele in healthy individuals is relatively low [5–7, 14]. The low frequency of the 1858T allele in this study may be one of the causes of the lack of a statistically significant association between the PTPN22 C1858T polymorphism and T1DM, because it limits the power of the study. The low frequency of the minor allele supports the need of a larger sample. Furthermore, the purpose of this study becomes more obvious. As a result, published data on the role of the PTPN22 C1858T polymorphism in individuals of Greek origin with TD1M are required. Until today, preliminary unpublished data of association studies in two patient populations of Greek origin with T1DM did not confirm the well-documented association of PTPN22 C1858T polymorphism with T1DM [38] in complete agreement with this study. Our data are the first published, and, although they imply a probable association, they indicate the need for a larger sample. However, the lack of association between PTPN22 C1858T polymorphism and T1DM in children and adolescents of Greek origin retains its research value, taking into account the geographical variation in the frequency of the 1858T allele, the genetic diversity of individuals, and the multifactorial nature of T1DM. Moreover, for the interpretation of our results it should take into account the different genetic background of populations concerning HLA class II genes as well as the variable effect of known or unknown predisposing environmental factors.

Although T1DM is an autoimmune disease without gender differentiation in prevalence, we found a tendency towards higher frequency of C1858T and T1858T genotypes in males. The lack of statistically significant association of the PTPN22 C1858T polymorphism with gender agrees with the majority of studies [7, 12, 13, 18, 25, 27]. In regard to the relationship of PTPN22 C1858T polymorphism with T1DM, some studies have suggested a gender differentiation in prevalence in favour of females [8, 10, 15, 16] and only one in favour of males [18]. While a few studies revealed a statistically significant association of the PTPN22 C1858T polymorphism with gender, a recently published meta-analysis suggests that males who carried the 1858T allele were more susceptible to T1DM than females [44]. As a result, the interaction of the PTPN22 C1858T polymorphism with gender in TD1 M requires further investigation.

It has been reported that genetic factors may influence the age of diagnosis. In this context, it was examined whether predisposing 1858T allele is associated with the age at diagnosis of T1DM. The mean age at diagnosis in 1858T allele carriers was similar to those of the remaining patients, in agreement with the conclusions of other researchers [8, 12, 13, 21, 27]. The results in terms of the possible effect of PTPN22 C1858T polymorphism in age of diagnosis are controversial, as it was associated with lower age of diagnosis [16] in 2 studies and particularly in the group of female patients [10, 15].

Taking into account the fact that autoimmune diseases accumulate in individuals and families, we studied the possibility of association of PTPN22 C1858TT polymorphism with personal history of HT and family history of T1DM without positive results in the study population. Recently published meta-analysis suggests association of the PTPN22 C1858T polymorphism with HT [33]. However, only 2 studies revealed a statistically significant association with the concordance of HT and T1DM [7, 9]. Therefore, further investigation is required—particularly in children and adolescent population—for the combined effect of the PTPN22 C1858T polymorphism and other factors in HT onset in patients with TD1 M. We investigated the contribution of the PTPN22 C1858T polymorphism to the aggregation of T1DM within families. The inability to find an increased frequency of the 1858T allele in patients with positive family history of T1DM is probably explained by the sample size.

In conclusion, the finding of increased frequency of the 1858T allele in patients with T1DM compared to healthy subjects agrees with the results of similar studies in other populations. The inability to detect statistically significant differences may be due to low frequency of the minor allele in Greek population. In order to make safe conclusions regarding the association of the PTPN22 C1858T polymorphism with T1DM in Greek population, a larger sample is needed because of the found rarity of the 1858T allele in Greeks.

## Figures and Tables

**Table 1:** The distribution of PTPN22 C1858T polymorphism in patients and controls.

	Patients (*N* = 130)	Controls (*N* = 135)
	Frequency (%)	
Genotype		
CC	116 (89.2)	127 (84.1)
CT	13 (10.0)	8 (5.9)
TT	1 (0.8)	0
* P*	0.273	
CT + TT	14 (10.8)	8 (5.9)
* P*	0.184	
Allele		
C	245 (94.2)	262 (97.0)
T	15 (5.8)	8 (3.0)
* P*	0.137	
OR	2.00 (CI 0.83–4.81)	

**Table 2:** The distribution of PTPN22 C1858T polymorphism in patients and controls according to gender.

	Males		Females	
	Patients (*N* = 73)	Controls (*N* = 72)	Patients (*N* = 57)	Controls (*N* = 63)
	Frequency (%)			
Genotype				
CC	64 (87.7)	67 (93.1)	52 (91.2)	60 (95.2)
CT	8 (11.0)	5 (6.9)	5 (8.8)	3 (4.8)
TT	1 (1.4)	0	0	0
*P*	0.416		0.475	
CT + TT	9 (12.3)	5 (6.9)	5 (8.8)	3 (4.8)
*P*	0.400		0.475	
Allele				
C	136 (93.2)	139 (96.5)	109 (95.6)	123 (97.6)
T	10 (6.8)	5 (3.5)	5 (4.4)	3 (2.4)
*P*	0.269		0.483	

**Table 3:** The distribution of PTPN22 C1858T polymorphism in patients with T1DM according to studied parameters.

	Allele		Genotype			
	C	T	CC	CT	TT	CT + TT
	Frequency (%)					
Gender						
(male/female) (73/57)	136/109 (93.2/95.8)	10/5 (6.8/4.4)	64/52 (87.7/91.2)	8/5 (11.0/8.8)	1/0 (1.4/0)	9/5 (12.3/8.8)
*P*	0.436		0.613			0.580
Age at onset						
(≤10/>10 years) (88/42)	166/79 (94.3/94)	10/5 (5.7/6)	79/37 (89.8/88.1)	8/5 (9.1/11.9)	1/0 (1.1/0)	9/5 (10.2/11.9)
*P*	1.000		0.701			0.769
Tanner stage at diagnosis						
(I/II–V) (99/31)	188/57 (94.9/91.9)	10/5 (5.1/8.1)	90/26 (90.9/83.9)	8/5 (8.1/16.1)	1/0 (1.0/0)	9/5 (9.1/16.1)
*P*	0.361		0.373			0.320
Ketoacidosis at onset						
(+/−) (104/26)	195/50 (93.8/96.2)	13/2 (6.3/3.8)	92/24 (88.5/92.3)	11/2 (10.6/7.7)	1/0 (1.0/0)	12/2 (11.5/7.7)
*P*	0.742		0.796			0.735
HT						
(+/−) (27/103)	49/196 (90.7/95.1)	5/10 (9.3/4.9)	23/93 (85.2/90.3)	3/10 (11.1/9.7)	1/0 (3.7/0)	4/10 (14.8/9.7)
*P*	0.206		0.141			0.488
Familiar history of T1DM						
(+/−) (19/111)	37/208 (97.4/93.7)	1/14 (2.1/6.3)	18/98 (94.78/88.3)	1/12 (5.3/10.8)	0/1 (0/0.9)	1/13 (5.3/11.7)
*P*	0.704		0.689			0.692
